# The future is in the background: background EEG patterns, not acute seizures, predict epilepsy and neurodevelopmental outcomes in neonatal HIE

**DOI:** 10.3389/fped.2025.1560760

**Published:** 2025-09-29

**Authors:** Kristine E. Woodward, Pauline de Jesus, Kimberly Amador, Pauline Mouches, Marvin Braun, Khorshid Mohammad, Nils D. Forkert, Michael J. Esser

**Affiliations:** ^1^ Department of Pediatrics, Cummings School of Medicine, University of Calgary, Calgary, AB, Canada; ^2^ Department of Clinical Neurosciences, Cummings School of Medicine, University of Calgary, Calgary, AB, Canada; ^3^ Department of Radiology, Cummings School of Medicine, University of Calgary, Calgary, AB, Canada; ^4^ Department of Electrical and Software Engineering, Schulich School of Engineering, University of Calgary, Calgary, AB, Canada

**Keywords:** neonatal HIE, cEEG = continuous EEG, neurodevelopmental outcome, epilepsy prediction, background EEG, background EEG activity

## Abstract

**Background:**

Hypoxic ischemic encephalopathy (HIE) is the most common neurologic emergency in the neonatal population, with a broad spectrum of potential neurodevelopmental outcomes. Additionally, HIE is the most common cause of seizures during the acute neonatal period. Unfortunately, predicting neurodevelopmental outcomes and epilepsy risk is difficult in this population, and seizure burden during the acute period has not consistently been correlated with outcomes in prior studies. We aimed to examine EEG background data to determine whether there is a relationship between background abnormalities, neurodevelopmental outcomes, and epilepsy risk, and whether this information is more informative for predicting outcomes compared to other clinical data points.

**Methods:**

Patients were retrospectively recruited from level 3 Neonatal Intensive Care Units (NICU's) in Calgary, Alberta, from 2014 to 2020. All patients who met the criteria for therapeutic hypothermia after being classified as at risk for HIE were included in the study. Clinical information captured included measures from clinical examination, blood work, MRI (day 3–5, scored using Barkovich scoring system) and medications. Continuous video EEG (cvEEG) recordings were separated into day 1, 2, and 3, and separate classifications systems were used for background and ictal findings. Neurodevelopmental follow-up was completed at two years of age, and patients were also categorized as having no epilepsy, or either well-controlled or refractory epilepsy. Poisson regression models and relative risk were used to compare background and ictal scores to long term neurodevelopmental outcomes and future epilepsy risk. Three supervised learning algorithms were trained to predict neurodevelopmental outcomes based on clinical factors.

**Results:**

Two-hundred and six patients were eligible for the study. Among neonates with seizures, only 18% developed epilepsy, while 52% of those with severely abnormal EEG background patterns did. Total ictal burden was not significantly associated with epilepsy at follow up, and no antiseizures medications were significant predictors. In contrast, EEG background score was strongly associated with epilepsy risk (adjusted *ß* = 2.75, *p* = 0.002), with severely abnormal backgrounds conferring significantly increased risk (37.5% vs. 5.2%, RR = 7.22, 95% CI: 3.09–16.88). Similarly, ictal burden did not predict poor neurodevelopmental outcome or death, whereas background score was a strong predictor (adjusted *ß* = 1.74, *p* < 0.001; RR = 2.44, 95% CI: 1.70–3.50). Machine learning models identified background features as more predictive than ictal scores, with XGBoost achieving the best classification performance (accuracy 0.724) and random forest yielding the highest AUC (0.751).

**Conclusions:**

In our cohort, EEG background patterns outperformed ictal burden in predicting both neurodevelopmental outcomes and future epilepsy risk. Although background patterns are not directly modifiable, they provide powerful, early markers of brain injury severity, offering clinicians a valuable tool for prognostication and family counseling at a critical juncture in care.

## Introduction

Hypoxic ischemic encephalopathy (HIE) is one of the most common neurologic emergencies in the neonatal population, occurring in 1–8 per 1,000 births worldwide (1). The spectrum of outcomes is broad, ranging from normal neurodevelopment to death. Developmental delays are common and can affect gross and fine motor skills, language and cognitive function, and social skills. Additionally, previous research suggests approximately 10% of patients develop epilepsy, many of whom are refractory to anti-seizure medications (2). Despite advances in neuromonitoring and targeted treatments during the acute phase, predicting patient outcomes to counsel families is difficult. Many studies have investigated potential predictors of long-term neurodevelopmental outcomes in HIE, with common variables being clinical examination (i.e., Sarnat score), specific laboratory measures (i.e., cord blood gas, lactate), neuroimaging findings (specifically MRI), and electroencephalography (EEG) tracings. With more widespread access to continuous EEG monitoring, research has expanded towards characterizing seizure burden and temporal EEG evolution in neonatal HIE, with many studies attempting to use these findings to help predict developmental outcomes. While some studies have described EEG characteristics and outcomes (3, 4), some with limited associations (5), others have identified promising early EEG predictors (6–11). The methodology has varied in using continuous EEG as a predictive marker, including calculating total vs. hourly seizure burden, characterizing EEG patterns during the rewarming period only, separating ictal vs. background EEG features, and using spot EEG analysis vs. averaging more prolonged periods (6–11). Until recently, most studies using quantitative EEG measures were small and did not include additional clinical variables in predictive models.

In this study, we incorporated neurophysiological and clinical data from a large cohort of patients with neonatal HIE using machine learning models, to evaluate the predictive ability for long-term neurodevelopmental outcomes, including future epilepsy risk. In particular, we examined specific EEG markers of background and ictal activity to explore the relationship with these outcomes. Understanding the relationship between clinical markers and outcomes affords the opportunity to improve acute clinical decision making and better guide prognostic discussions with families.

## Materials and methods

### Patient cohort

Patients were retrospectively recruited from level 3 Neonatal Intensive Care Units (NICUs) in Calgary, Alberta from 2014 to 2020. All patients who met the criteria for therapeutic hypothermia (TH) after being classified as at risk for hypoxic ischemic encephalopathy (HIE) were included in the study. At our institution, initiation of therapeutic hypothermia requires first that babies are ≥35 weeks gestation and ≤6 h old, and subsequently meet both criteria A and B defined as follows: (A) umbilical cord or first-hour arterial gas pH ≤ 7.0 or base excess ≤ −16 (mmol/L), or Apgar score ≤5 at 10 min, or ongoing need for respiratory support at 10 min of birth; AND (B) evidence of moderate to severe encephalopathy, demonstrated by the presence of seizures or at least one sign in three or more of six major categories (Sarnat Score: level of consciousness, spontaneous activity, posture, tone, primitive reflexes, autonomic system) (12). Additionally, patients were excluded from our study if they were moribund or had any major congenital/genetic abnormalities for which no further treatment was planned, severe intrauterine growth restriction (IUGR), significant coagulopathy, or severe intracranial bleeding (12). Patients were also excluded if electronic medical records were not accessible to capture the variables listed below. Research was conducted in accordance with institutional requirements and policies (IRISS University of Calgary #REB15-1249).

### Clinical data

Babies categorized as having HIE were treated with therapeutic hypothermia using whole-body cooling blankets with a built-in thermoregulator (CritiCool®) that maintained a temperature of 33.5 degrees Celsius. Continuous EEG was recorded for the duration of TH (∼72 h) and until the babies were rewarmed to physiological normal temperature (∼6 h).

Clinical information was captured for each patient, including referral centre (rural, urban non-cooling centre, urban cooling centre), gestational age at birth, birth weight, APGAR score at 1, 5, and 10 min, Sarnat score at admission and discharge (13), cord arterial pH, cord arterial base excess, lactate at one hour of age, anti-seizure medications (ASMs) administered acutely (e.g., levetiracetam, phenobarbital, fosphenytoin), and if continued upon discharge, pain/sedative medications (morphine, dexmedetomidine, fentanyl) administered, MRI (day 3–5), EEG (72 h recording), length of stay, a diagnosis of epilepsy at out-patient follow-up, and neurodevelopmental follow-up assessments as described below.

Magnetic resonance imaging scans (1.5 T or 3 T Siemens MR Scanner) were graded based on the combined Barkovich basal ganglia/watershed scoring system by a neuroradiologist and pediatric neurologist using T1- and T2-weighted images (14). EEGs were scored by two independent neurophysiologists (KW and MB). EEG data capture (Natus® NeuroWorks®, restricted 10–20 system using nine electrodes and designated neonatal montage) was separated into day 1 (from initiation of recording to 24 h since cooling onset), day 2 (24 to 48 h of cooling), and day 3 (48 to 72 h of cooling). Background scores and ictal scores were analyzed and calculated separately as follows using the American Clinical Neurophysiology Society Standardized EEG guidelines for Neonates (15). For background scores, a score of 0 indicated normal continuity whereby there was uninterrupted non-stop electrical activity with <2 s of voltage attenuation <25 uV. A score of 1 indicated abnormal excessive discontinuity, where the IBI was prolonged or voltage depressed (for term, longer than 6 s and <25 uV). Severely abnormal background (score of 2) indicated invariant, abnormally composed EEG bursts (or no bursting) with low voltage <5 uV and no normal electrographic elements within the bursts. Additionally, seizure burden was calculated for each patient during day 1, 2 and 3 for both the entire 24 h, as well as highest 1 h seizure burden period during that day (using a sliding-window technique). Total ictal burden was calculated for the entire recording for each patient (as a continuous variable). When specified, the highest 1 h seizure burden was used to provide an “ictal score” for each day, whereby 0 = no seizures, 1 = seizures but not meeting criteria for status epilepticus, and 2 = status epilepticus (≥30 min in 1 h). Total background score was calculated by adding each daily score for a score out of 6 (i.e., worst score would be severe suppression on day 1, 2 and 3 = 2 + 2 + 2 = 6, and best score would be 0, equating a normal background for all three days), and total ictal score was calculated by adding each daily score for a score out of 6 (i.e., worst score would be status epilepticus on day 1, 2, and 3 = 2 + 2 + 2 = 6, and best score would be 0, indicating no seizures). Neurodevelopmental follow-up was completed at approximately 24 months using the Ages and Stages Assessment (16). Neurodevelopmental impairment was characterized as ≥2 standard deviations outside the normal range in any domain.

### Statistical analysis

Each feature was compared to neurodevelopmental outcomes using a Mann–Whitney *U*-test, and Benjamini–Hochberg correction for false discovery rate. We used Poisson regression models with restricted cubic splines to evaluate the relationship between total EEG background score and neurodevelopmental outcome, as well as future epilepsy risk. The primary outcome was binary (poor vs. good neurodevelopmental outcome, or epilepsy vs. no epilepsy) and the main predictor was total EEG background score. Splines were used to flexibly model non-linear relationships without assuming a specific parametric form. We included both an unadjusted model (included only the spline-transformed EEG background score as a predictor) and an adjusted model [including binary indicators for medication exposure (dexmedetomidine, morphine, fentanyl, levetiracetam, phenobarbital, fosphenytoin)]. Poisson models were fit using the Generalized Linear Model framework with a log link function. Model fit was assessed using pseudo *R*^2^ statistics (Cragg-Uhler), and 95% confidence intervals were generated for all predictions. Predictor probabilities of poor neurodevelopmental outcome, or epilepsy, were plotted against EEG background scores. Based on these results, patients were split into two groups; those with “severely abnormal EEG background scores (total score of 5 or 6)” and those with “mildly/moderately abnormal or normal EEG background scores (total score of 0–4)”. Relative risk, using a 95% confidence interval, was calculated to determine risk of future epilepsy. The same was used to calculate risk of poor neurodevelopmental outcomes or death.

Ictal scores were also compared to both neurodevelopmental follow up and future epilepsy risk using Poisson regression models with restricted cubic splines, and relative risk was determined as described above.

Chi-squared test was used to calculate the difference in future epilepsy between patients that were discharged on ASMs and those who were not.

SPSS and Python were used to conduct all statistical analyses.

### Machine learning setup

The machine learning paradigm used in this study consisted of a feature ranking and selection method followed by a classification model. The aim of feature ranking is to sort the available features based on relevance or importance to predict the outcome variable (i.e., good/normal vs. poor/abnormal neurodevelopmental outcome), which is then used for feature selection (17–19).

For this study, the information gain algorithm was used to statistically determine the amount of information that is gained from each feature when predicting neurodevelopmental outcomes. The resulting feature ranking was then used to determine the optimal number of input features for the classifier. This was achieved by removing the least relevant features in an iterative fashion and then retraining and evaluating the classifier in terms of accuracy, thereby decreasing the dimensionality problem to improve the model performance. Three supervised learning algorithms were used to evaluate predictive performance, including logistic regression, random forest, and extreme gradient boosting, in order to account for imbalance and smaller datasets.

The machine learning models were trained to predict good/normal vs. poor/abnormal neurodevelopmental outcomes. All of the clinical features outlined above were initially included in the model and iteratively reduced by removing the lowest-ranked feature. Due to the class imbalance between normal and abnormal neurodevelopmental outcomes, a random under-sampling approach was used, resulting in a perfectly balanced dataset. We repeated this process ten times to reduce any potential bias induced by the random under-sampling approach.

Using the balanced datasets, a 10-fold cross-validation approach was used to quantitatively evaluate the model performance. This means that ten different models were trained for each experiment, each of which randomly selected 90% of the data for training and 10% for testing. The results of each fold were averaged to compute outcome measures, including accuracy (i.e., percent correctly classified), precision (i.e., positive predictive value), recall (i.e., sensitivity), F-measure (i.e., harmonic mean of precision and recall), and area under the curve.

## Results

### Patient characteristics

Two-hundred and six patients were admitted to hospital between 2014 and 2020 and were eligible for the study. Patient information is listed in Table 1, including median, interquartile ranges, minimum, and maximum values for each clinical variable for each outcome group (normal neurodevelopmental outcome vs. poor neurodevelopmental outcome or death). At admission to the NICU, clinical examinations were documented, and patients were classified as mild, moderate, or severe HIE based on the Sarnat classification scale. For comparison, clinical examinations were also documented after rewarming using the Sarnat classification scale. Most patients had lower Sarnat scores post rewarming, and no patients had higher scores.

**Table 1:** Summary of patient data. Variables shown in column 1, Column 2 and 3 depict patients separated into groups of normal neurodevelopmental outcome and poor neurodevelopmental outcome/death with medians, and interquartile ranges in brackets. Column 4 depicts the minimum and maximum score for each variable in the total patient group. Column 5 shows the *p*-value comparing groups of normal and poor neurodevelopmental outcome/death for each variable using Mann–Whitney *U*. The last column shows the corrected *p*-value for multiple comparisons using the Benjamini–Hochberg method.

Variable	Normal neurodevelopmental outcome group [median (IQR)]	Poor neurodevelopmental outcome or death group [median (IQR)]	Min–max (all patients)	Raw *p*-value	BH adjusted *p*-value
Birth gestational age	39.50 (38.53–40.29)	39.22 (37.29–40.57)	35.00–42.43	0.328	0.420
Birth weight	3.34 (2.97–3.66)	3.37 (2.91–3.99)	1.25–33.60	0.395	0.478
Arterial pH	6.97 (6.86–7.11)	6.99 (6.80–7.15)	6.30–7.37	0.857	0.896
Arterial base excess	−15.00 (−19.00–−10.00)	−13.50 (−23.00–−8.75)	−30.00–−0.30	0.940	0.940
Lactate	10.30 (6.35–13.90)	11.70 (6.15–17.95)	−10.00–21.00	0.185	0.304
APGAR 1 min	2.00 (1.00–3.00)	1.00 (0.00–2.00)	0.00–9.00	0.031	0.071
APGAR 5 min	4.00 (3.00–5.75)	3.50 (2.00–6.00)	0.00–9.00	0.589	0.678
APGAR 10 min	6.00 (4.00–7.00)	5.00 (3.00–7.00)	0.00–10.00	0.316	0.420
MRI Results	0.00 (0.00–3.25)	2.50 (0.00–5.00)	0.00–5.00	0.030	0.071
Neuro exam at admission	2.00 (1.00–2.00)	2.00 (2.00–3.00)	0.00–3.00	0.003	0.015
Neuro exam post re-warm	0.00 (0.00–0.00)	0.00 (0.00–3.00)	0.00–3.00	0.028	0.071
Background day 1	0.00 (0.00–1.00)	1.00 (0.00–2.00)	0.00–2.00	0.008	0.049
Background day 2	0.00 (0.00–1.00)	1.00 (0.00–2.00)	0.00–2.00	0.002	0.035
Background day 3	0.00 (0.00–1.00)	1.00 (0.00–2.00)	0.00–2.00	0.004	0.035
Total background score	0.00 (0.00–3.00)	3.00 (0.00–6.00)	0.00–6.00	0.004	0.035
Ictal day 1	0.00 (0.00–0.25)	0.00 (0.00–0.75)	0.00–2.00	0.765	0.838
Ictal day 2	0.00 (0.00–0.00)	0.00 (0.00–0.00)	0.00–2.00	0.201	0.308
Ictal day 3	0.00 (0.00–0.00)	0.00 (0.00–0.00)	0.00–2.00	0.024	0.071
Total ictal score	0.00 (0.00–1.00)	0.00 (0.00–1.00)	0.00–6.00	0.328	0.420

MRIs were available for 196 patients, with 10 not performed due to patient death prior to imaging. Five patients had MRIs on day 3 immediately preceding death, whereas the remainder were scanned on day 4 or 5. One-hundred and thirty-eight patients had normal MRIs (70.4%). Of the remaining patients, 11 had a Barkovich score of 1 (5.6%), 16 had a score of 2 (8.2%), 19 had a score of 3 (9.7%), and 12 had a score of 4 (6.5%).

**Figure 1:**
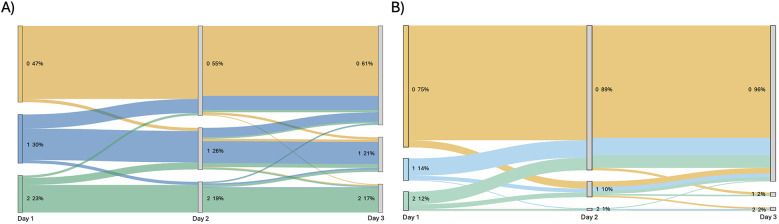
Sankey diagram showing **(A)** change in background EEG score over three days of recording for each patient and **(B)** change in ictal EEG score over three days of recording for each patient. Score is beside each node, with percentage of total patients with that score shown beside.

**Figure 2:**
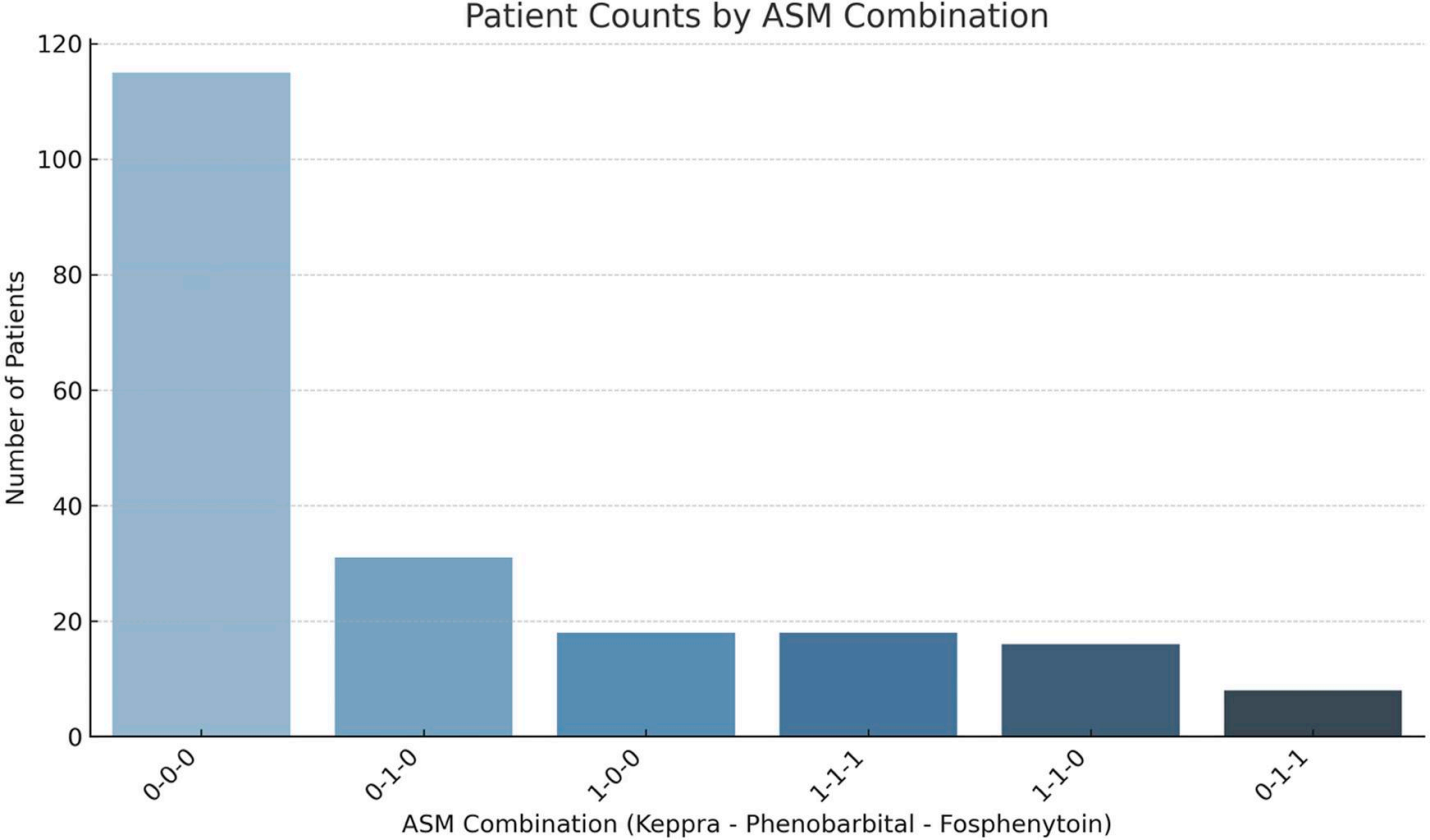
Number of patients that received each combination of anti-seizure medications.

EEG results were available for 191 patients on all three days. Background and ictal scoring results are shown in Figure 1. Background patterns overall, even in moderate and severe HIE, showed a trend towards normalization from day 1 to 3, with an increasing number of EEGs receiving a score of 0, and a decreasing number of EEGs with a score of 1 or 2. In terms of ictal grading, there was increasingly more patients with a score of 0 (i.e., no ictal activity) over the three days. However, only 4% patients continued to have seizures on day 3. Also of importance, only 1.5% of patients with seizures on day 3 did NOT have seizures on either day 1 or day 2. Thirty four percent of patients had seizures on EEG during the first three days of life. In terms of total ictal burden, patients on average had 53 min and 30 s of ictal EEG activity over the three days (range: 0 h–43 h 24 min). For day 1 this was a mean of 0 h 35 min 39 s (range: 0 h–14 h 25 min), for day 2 this was a mean of 0 h 11 min 19 s (range: 0 h–19 h 15 min), and for day 3 this was a mean of 0 h 6 min 3 s (range: 0 h–9 h 44 min). Forty-eight percent of patients received anti-seizure medications; of these, 54% had abnormal movements suspected to be clinical seizures prior to EEG being connected, without any further seizures on EEG. The most frequently used anti-seizure medication was phenobarbital, followed by levetiracetam and then fosphenytoin (further details shown in Figure 2).

From the total cohort, 7.3% patients died during the acute period in the hospital. An additional 6.3% were lost due to missing follow-up information at 24 months. At follow-up, 37.1% had neurodevelopmental impairment in at least one domain according to the Ages and Stages Assessment, and 62.9% had normal neurodevelopment at 24 months. Patients were separated into good/normal vs. poor/abnormal overall as a binary measure, given the low power in separating them based on each abnormal neurodevelopmental domain or feature.

**Figure 3:**
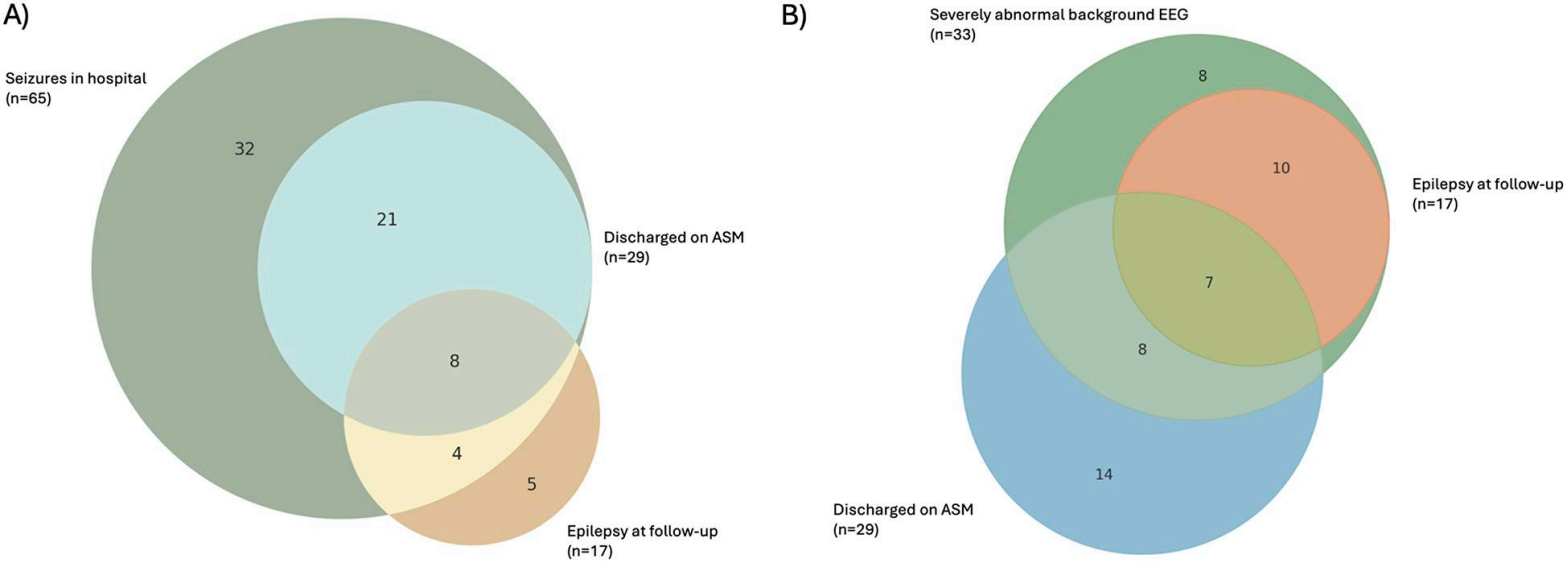
Weighted venn diagrams showing number of patients with overlap, having **(A)** seizures in hospital, being discharged on ASMs, and having epilepsy at follow-up or **(B)** having a severely abnormal background EEG, being discharged on ASMs, and having epilepsy at follow-up. Numbers in each overlap area indicate number of patients falling into that overlap region. N in brackets depicts total number of patients in that category.

Sixty-five patients (34%) had EEG confirmed seizures at some point during the acute period, and of these, 12 patients with seizures in the hospital developed epilepsy (18.5%). Five patients without seizures in hospital developed epilepsy (4% of patients without acute seizures). Seventeen patients (8.9%) had epilepsy, and 8 (4.2%) of these patients had refractory epilepsy at follow-up. Of the 17 patients with epilepsy, 8 were discharged from the hospital on anti-seizure medications (47.0% of those with epilepsy at follow up). In total, 29 patients were discharged on anti-seizure medications due to physician guidance (potentially for parental preference), all of whom had seizures while in the hospital. Twelve of the patients with epilepsy at follow-up had seizures in the hospital (70.6% of the patients with epilepsy). See Figure 3 for a pictorial depiction.

Severely abnormal EEG background classification was given for those babies with a total background score of 5 or 6. There were 33 patients with a background score of 5 or 6 during the 3 days of EEG recording. Of these 33 patients, 17 had epilepsy at follow up (51.5%), 7 of whom were discharged on ASMs. See Figure 3 for a pictorial depiction.

Within our cohort, there were no significant differences in epilepsy prevalence between the groups who were discharged on ASM and those who were not (*X*^2^ = 1.68, *p* = 0.19).

### Future epilepsy risk

As mentioned above, only 18.5% of patients with seizures in hospital had epilepsy at follow up. In contrast, 51.5% of patients with severely abnormal EEG background scores had epilepsy at follow up.

**Figure 4:**
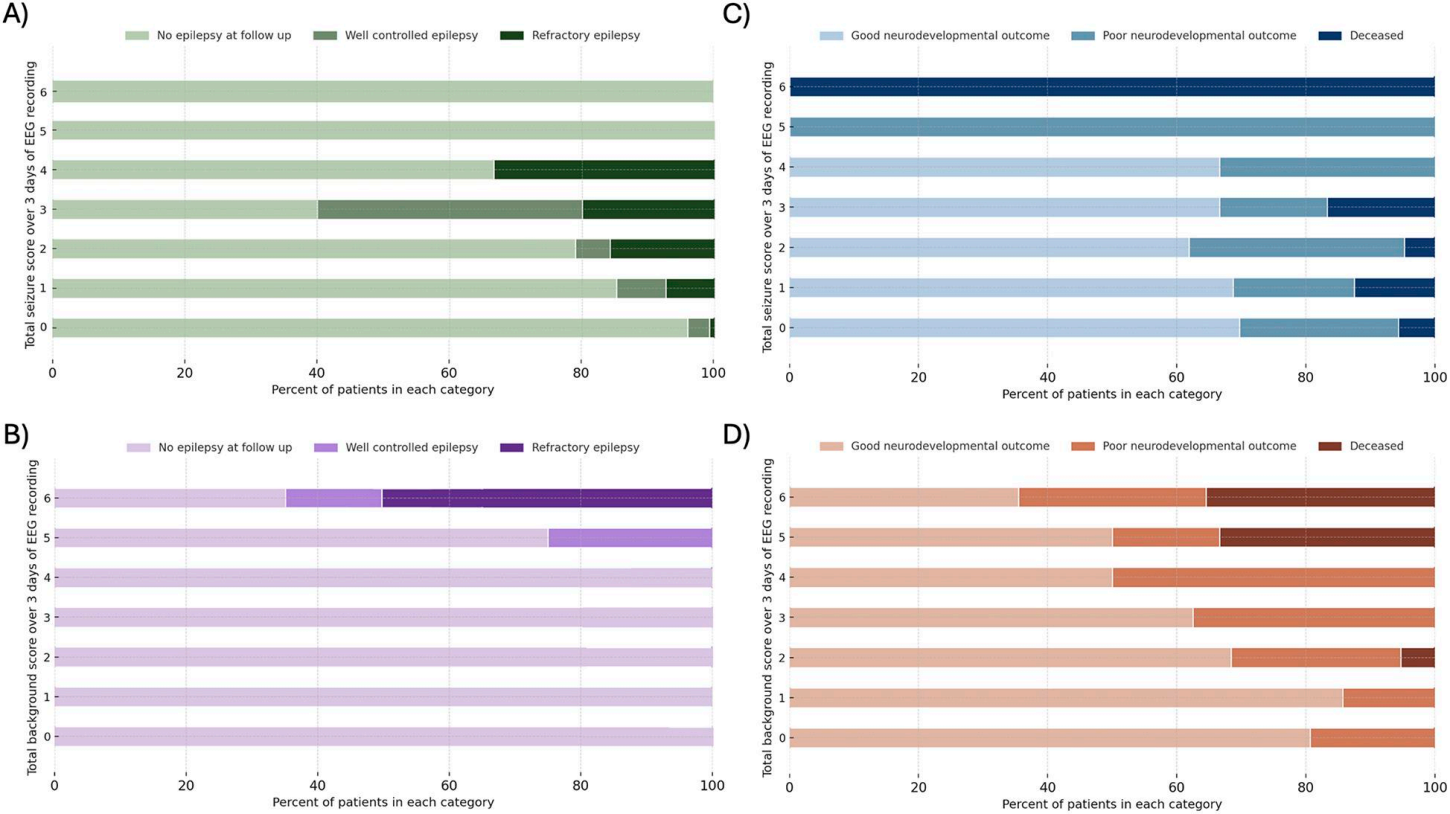
Comparison of patient groups based on **(A)** total 3-day ictal scores and **(B)** total 3-day background scores and epilepsy at follow-up, including patients with no epilepsy, those with well controlled epilepsy, and those with refractory epilepsy; and comparison of patient groups based on **(C)** total 3-day ictal score and **(D)** total 3-day background scores and outcomes at follow-up, including patients with good neurodevelopmental outcomes, those with poor neurodevelopmental outcomes, and patients who died in hospital.

Figure 4 depicts the distribution of patients with no epilepsy, well controlled epilepsy, and refractory epilepsy at follow up in groups of patients separated based on total ictal scores while in hospital. As shown, there is no clear trend to suggest a relationship between worse ictal scores and epilepsy. In line with this, using a Poisson regression model with restricted cublic splines there was not a significant association between total ictal burden and epilepsy at follow up (*ß* = −0.0002, *p* = 0.47). None of the covariates (levetiracetam, phenobarbital, fosphenytoin, morphine, fentanyl, dexmedetomidine) demonstrated statistically significant associations with epilepsy risk. This model explained approximately 2% of the variance in the epilepsy outcome (pseudo *R*^2^ = 0.0196). This was also analyzed without ASMs as covariates, and in the unadjusted Poisson regression model, total ictal burden was not significantly associated with epilepsy at follow up (*ß* = −0.0002, *p* = 0.43), with pseudo *R*^2^ = 0.011 indicating that the ictal burden alone explains approximately 1% of the variability in epilepsy outcomes. Calculating relative risk, patients with seizures in hospital were not more likely to have epilepsy at follow-up compared to those without seizures in hospital (18% vs. 4% RR = 3.25, 95% CI = 0.759–13.907).

**Figure 5:**
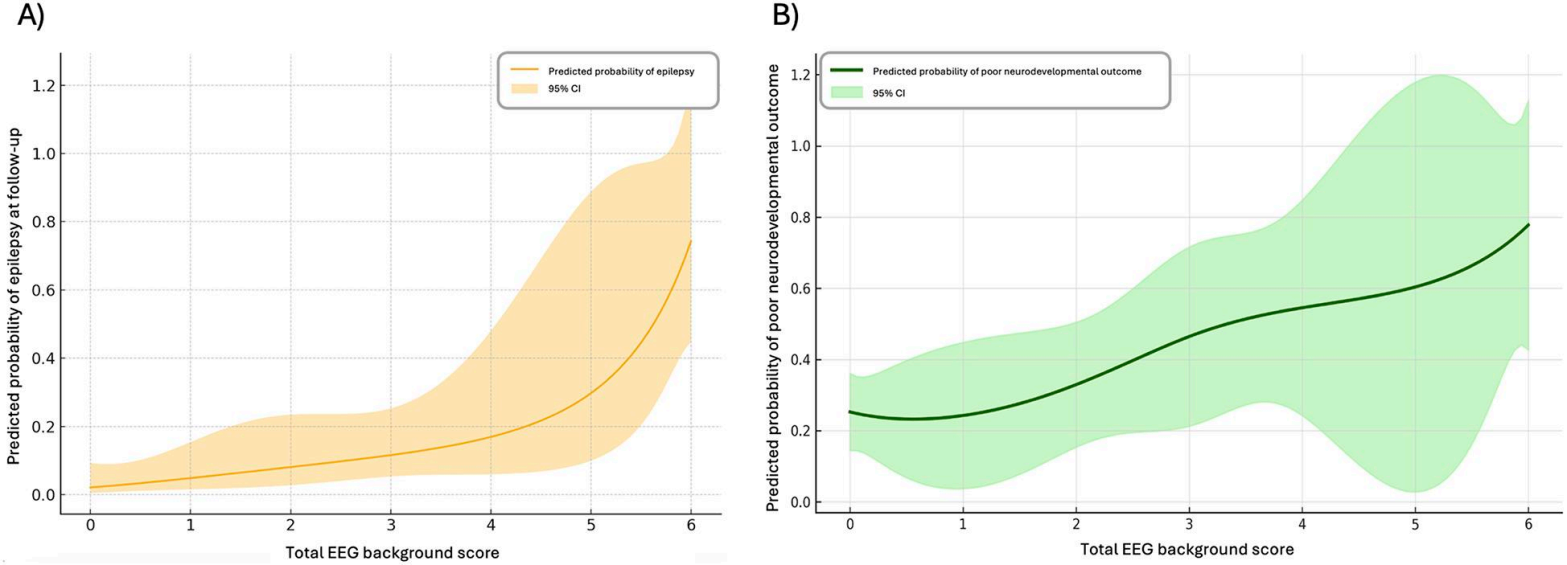
Poisson regression models with restricted cubic splines and 95% confidence intervals for **(A)** predicted probability of epilepsy at follow-up based on total EEG background scores over 3 days and **(B)** predicted probability of poor neurodevelopmental outcome or death based on total EEG background scores over 3 days.

Figure 4 also shows the number of patients with no epilepsy, well-controlled epilepsy, and refractory epilepsy at follow up in groups of patients based on total background score while in hospital. As can be seen, there is a trend to suggest a relationship between worse background scores and likelihood of having epilepsy. In line with this, using a Poisson regression model with restricted cubic splines (df = 3) we identified a non-linear relationship, with a steep increase in epilepsy probability observed among patients with more severe background abnormalities (Figure 5). This suggests that while mildly abnormal backgrounds display similar low future epilepsy risk, severely abnormal EEGs are particularly predictive of epilepsy (spline 3; *ß* = 3.55, *p* < 0.001) and the spline-based model explains 34.5% of the variance in epilepsy outcome (pseudo *R*^2^ = 0.345). Importantly, this association persisted after adjusting for antiseizure medications (*ß* = 2.75, *p* = 0.002 and pseudo *R*^2^ = 0.39). Phenobarbital and levetiracetam were independently associated with higher probability of epilepsy, likely due to clinical indication in that they are used in patients at higher clinical risk. No other medications had significant association with epilepsy (fosphenytoin, morphine, fentanyl, dexmedetomidine). Given these findings, patients with severely abnormal background scores (5 or 6) were compared to those with mildly abnormal or normal background scores (0–4), and using relative risk ratios were found to have significantly increased risk of epilepsy at follow-up [51.5% vs. 0% RR = 2.13 (adjusted by applying a continuity correction given the “0%”), 95% CI = 1.47–3.07].

### Neurodevelopmental outcomes

Mann–Whitney *U*-test for each feature compared to neurodevelopmental outcome, corrected for multiple comparisons **(**Benjamini–Hochberg), is listed in Table 1. The only significant features were neurological exam at admission, and background EEG scores.

Average total ictal scores compared with neurodevelopmental outcomes and death are depicted in Figure 4. No clear trend was apparent. The poisson regression model with restricted cubic splines did not demonstrate a significant relationship between total ictal burden and outcomes (*ß* = 0.0002, *p* = 0.43, pseudo *R*^2^ = 0.011). Results did not change taking medications into account as a covariate. When comparing patients with any seizures in hospital compared to those with none, there was not an increased risk of poor neurodevelopmental outcomes/death (42% vs. 33% RR = 1.339, 95% CI = 0.911–1.968).

Figure 4 demonstrates the number of patients in each neurodevelopmental outcome category with groups of patients separated based on total background score while in hospital. As shown, there is a trend to suggest a relationship between worse background scores and poor outcomes. Using a Poisson regression model with restricted cubic splines (df = 4), there was a significant association between total background score and neurodevelopmental outcome, using two models (both adjusted and unadjusted for medications) (Figure 5). In the unadjusted model, higher EEG background scores were associated with increased risk of poor neurodevelopmental outcome (*ß* = 1.55, *p* < 0.001), with the model explaining a moderate amount of outcome variability (pseudo *R*^2^ = 0.183). In the adjusted model (including medications as covariates), similar findings were seen, with higher splines showing significant associations with outcome (*ß* = 1.74, *p* < 0.001), with similar pseudo *R*^2^ values (0.189). Patients with severely abnormal background scores were more likely to have poor neurodevelopmental outcomes than those with good background scores while in hospital (62.2% vs. 25.4% RR = 2.44, 95% CI = 1.70–3.50).

### Predictive modelling

**Figure 6:**
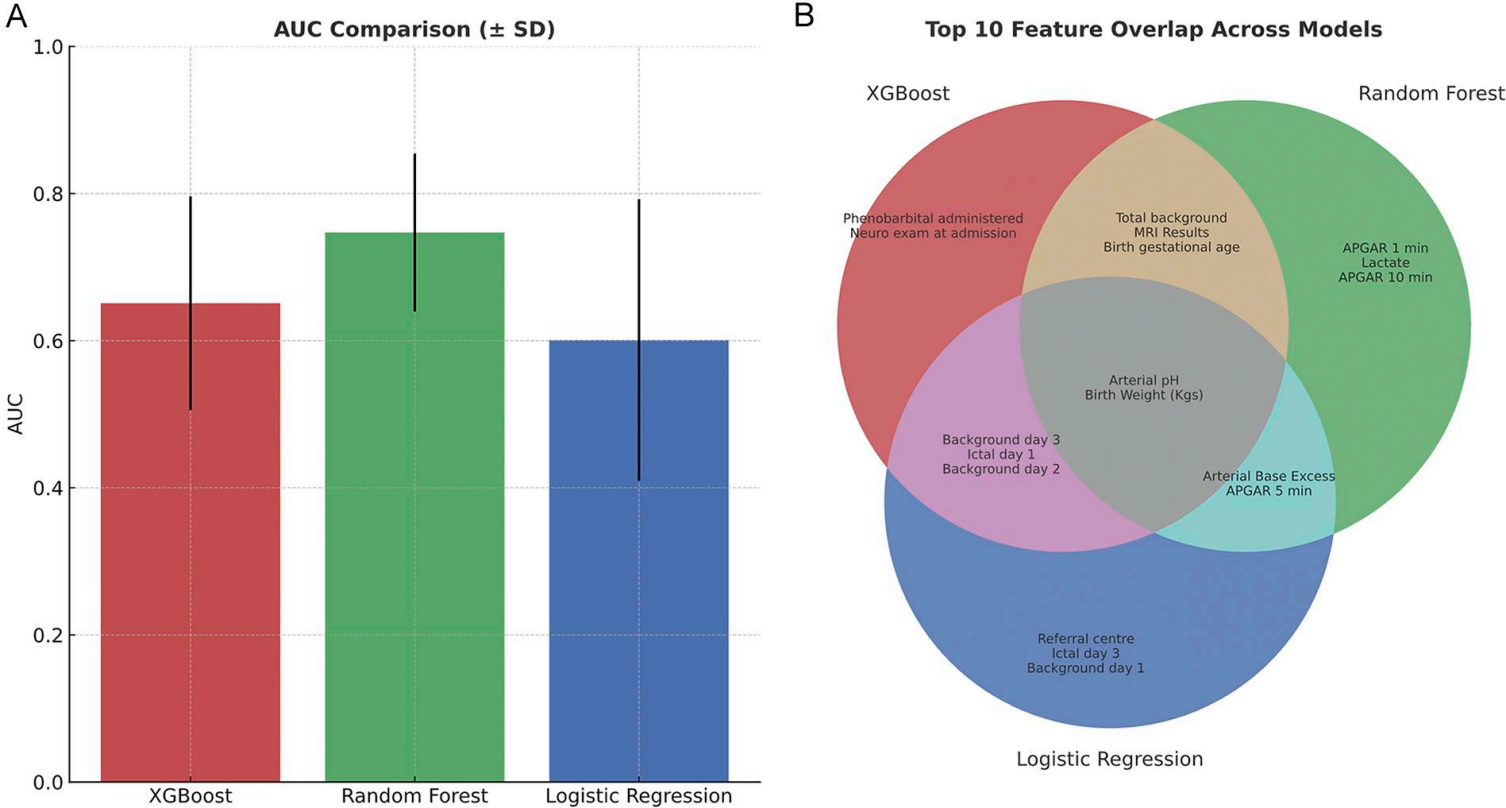
**(A)** Comparison of all three machine learning models area under the curves (AUC) for predicting good vs. poor neurodevelopmental outcome/death and **(B)** the top ten features selected for each model, and where they overlapped between models.

Model performance varied across the three classifiers. XGBoost achieved the highest accuracy (0.724), precision (0.611), recall (0.476), and F1 score (0.519), indicating superior performance in identifying cases with poor outcomes (Figure 6, Table 2). However, random forest had the highest AUC (0.751) suggestion better overall discrimination ability (Figure 6, Table 2). To better illustrate overlap in predictive value across methods, a Venn diagram was constructed comparing the top 10 features selected by each of the three models (Figure 6). Interestingly, arterial pH and birth weight were selected in all three models as important features. Background scores were represented more frequently in overlap sections compared to ictal scores, in line with previous results. Other important features in multiple models included MRI scores, gestational age, arterial base excess and APGAR score at 5 min.

**Table 2:** Model performance across the three supervised learning algorithms for predicting poor neurodevelopmental outcome or death.

Model	Accuracy	Precision	Recall	F1 score	AUC
Random Forest	0.685	0.584	0.321	0.383	0.751
Logistic Regression	0.680	0.497	0.352	0.403	0.685
XGBoost	0.724	0.611	0.476	0.519	0.651

AUC, area under the curve.

## Discussion

This large cohort study examined the importance of specific clinical factors in predicting both neurodevelopmental outcomes and future epilepsy risk in patients with neonatal HIE.

Our main findings demonstrated:

1. cvEEG is an important predictor of neurodevelopmental outcomes.
2. cvEEG background scores are stronger than ictal burden at predicting mortality and poor neurodevelopmental outcomes.
3. cvEEG background scores are stronger than ictal burden at predicting epilepsy at follow up.
4. ASM use in hospital and at discharge does not correlate with future epilepsy risk.

Understanding risk factors for poor neurodevelopmental outcomes and epilepsy can help guide acute treatment decisions, aid in early prognostication, and lead to more informed patient care.

### Neurodevelopmental outcomes

In our study, neurodevelopmental outcomes and in hospital mortality were significantly associated with EEG background, but not EEG ictal scores. In 2023, nested within the RCT HEAL study, Glass et al. also reported a significant association between EEG background scores in hospital and neurodevelopmental outcomes (20). The reproducibility of these results with large numbers, but at a single centre, reinforces the importance of these findings. Figure 4 shows that the highest percentage of deceased patients in the group had background EEG scores of 5 or 6. Further, while more patients had suppressed backgrounds on day 1 (Figure 1), these improved by day 3. In order to receive a score of 5 or 6, the background had to remain suppressed or discontinuous for the entirety of the 3 day recording. These results show the added utility of recording EEG for 3 days during the acute period to aid in predicting outcomes, but one could argue that this is predominantly necessary in babies where the EEG background is classified as ’severely abnormal' at the onset to ensure resolution.

### Seizure trends and future epilepsy risk

EEG ictal scores were not associated with neurodevelopmental outcomes or future epilepsy risk. Overall, seizure frequency decreased over the three days of cooling, except in a small (4%) percentage that continued to have seizures on day 3, reinforcing the limited need for maintenance ASMs in patients with HIE. A recent study by Glass et al. in 2021 (21) demonstrated the lack of evidence for continuing ASMs in patients with neonatal seizures after discharge from hospital. Our study is aligned with those findings, as there were no significant differences in rates of epilepsy at follow-up in patients discharged home on ASMs vs. those who were not. Furthermore, seizures in hospital did not predict epilepsy at follow-up. In fact, 5 patients with epilepsy at follow-up did not have acute seizures during their admission period, reinforcing the difficulty in predicting future risk of epilepsy.

Overall, in our cohort, EEG background scores were more predictive of epilepsy at follow up than ictal burden. This finding may be explained by the fact background abnormalities reflect global cerebral injury and neuronal dysfunction; severely abnormal EEG backgrounds, such as discontinuity or burst suppression, indicate widespread cortical damage and impaired synaptic recovery, thereby conditions that promote long-term epileptogenesis. In contrast, ictal burden primarily reflects transient instability in the acute phase, and not necessarily long-term neuronal damage and network reorganization. Therefore, background EEG scores, as suggested by our data, is likely a more stable and prognostically relevant biomarker for epilepsy risk in this population.

### Predictive modelling using machine learning

The comparative analysis of machine learning models highlights the clinical promise of data driven prediction in neonatal neurocritical care. Among the models tested, XGBoost demonstrated the strongest overall classification performance, suggesting higher sensitivity in detecting infants at risk of poor neurodevelopmental outcome or death. This model consistently identified EEG background scores (individual days and total), and electrographic seizures on day 1 as key predictors, aligning well with existing literature (6). Figure 6 illustrates the overlap in predictive value across methods for top ten features, and highlights how different algorithms capture distinct patterns in data. The multidimensional perspective reinforces the central importance of early EEG findings in prognostication, while also suggesting complementary contributions from metabolic (i.e., lactate, arterial BE) and clinical (i.e., referral center, birth weight) variables.

## Limitations

Despite the inclusion of a relatively large cohort, this study was affected by a class imbalance, with a smaller proportion of patients experiencing poor neurodevelopmental outcomes or death and lower rates of epilepsy at follow up. This imbalance may have limited the initial statistical analyses as well as the predictive performance of the machine learning models, particularly in accurately identifying high-risk patients. Increasing the representation of poor outcomes in future datasets may enhance model calibration and discrimination. A larger sample size would also support stratification of neurodevelopmental outcomes into specific cognitive and behavioral domains, which could reveal domain-specific vulnerabilities (e.g., executive function) when examined in conjunction with long-term follow-up data.

This analysis used neurological examinations from NICU admission, typically within the first 6 h of life, rather than immediate postnatal assessments. Due to documentation constraints, earlier clinical findings at the referring hospital may have influenced the decision to initiate therapeutic hypothermia, and patients may have shown clinical improvement or deterioration during transfer. This impacts the utility of using mild, moderate, and severe examination grouping in this dataset.

While anti-seizure medications can transiently alter EEG background features (e.g., increased discontinuity or voltage suppression), prior studies have shown EEG background typically recovers within 4 h of administration (22). Given that EEG background was assessed continuously over 24 h intervals, our analyses likely reflect stable background characteristics rather than short-term medication effects (22). Additionally, ASMs as well as sedative medications were used as covariates in multiple analyses as described above.

While our use of XGBoost and repeated sampling strategies was intended to enhance predictive performance, we acknowledge that these methods do not provide causal estimates of feature effects. If the primary objective were to estimate the independent effect of EEG background or ictal burden, methods such as inverse probability of treatment weighting (IPTW) or marginal structural models (MSMs) would be more appropriate and interpretable. Future work aiming to quantify causal effects should consider these approaches to complement the predictive modeling framework presented here.

Lastly, our models have not yet undergone external validation**,** which limits generalizability. Nonetheless, the results are consistent with findings from larger studies, supporting the robustness of the approach.

## Conclusions

Neonates with HIE present with a spectrum of clinical and EEG findings, from having no seizures to status epilepticus, and normal to severely abnormal backgrounds. As such, prognosis for short- and long-term outcomes can be challenging. Scoring EEGs using separate measures for background and ictal characteristics may serve to better predict patients with in- hospital mortality, poor neurodevelopmental outcomes, and future epilepsy risk. Results for our study suggest that severely abnormal EEG background scores are significantly associated with outcomes, whereas acute seizures at the time of presentation are not. Characterization of specific EEG patterns aids in understanding clinical progression, guiding treatment decisions, and providing families with earlier prognostication.

## Data Availability

The data analyzed in this study is available upon request. Requests to access these datasets should be directed to kristine.woodward@albertahealthservices.ca.
